# Triple Combinations of AAV9-Vectors Encoding Anti-HIV bNAbs Provide Long-Term In Vivo Expression of Human IgG Effectively Neutralizing Pseudoviruses from HIV-1 Global Panel

**DOI:** 10.3390/v16081296

**Published:** 2024-08-14

**Authors:** German A. Shipulin, Dina V. Glazkova, Felix A. Urusov, Boris V. Belugin, Valeriya Dontsova, Alexandra V. Panova, Alyona A. Borisova, Galina M. Tsyganova, Elena V. Bogoslovskaya

**Affiliations:** 1 Centre for Strategic Planning and Management of Biomedical Health Risks, Federal Medical Biological Agency, 119992 Moscow, Russialenabo2@mail.ru (E.V.B.); 2 Izmerov Research Institute of Occupational Health, 105275 Moscow, Russia

**Keywords:** HIV-1, broadly neutralizing antibodies, AAV9 vector, mice, N6, PGT128, PGDM1400, VCR07-523, 10-1074, 10E8

## Abstract

Anti-human immunodeficiency virus (HIV) broadly neutralizing antibodies (bNAbs) offer a promising approach for the treatment of HIV-1. The current paradigm for antibody therapy involves passive antibody transfer, requiring regular delivery of bNAbs in treating chronic diseases such as HIV-1. An alternative strategy is to use AAV-mediated gene transfer to enable in vivo production of desirable anti-HIV-1 antibodies. In this study, we investigated two sets of triple combinations of AAV9-vectors encoding different bNAbs: N6, 10E8, 10-1074 (CombiMab1), and VRC07-523, PGDM1400, 10-1074 (CombiMab2). We used CBAxC57Bl and C57BL/6 mouse models to characterize rAAV-induced antibody expression and to evaluate the neutralization capacity of mouse sera against a global panel of HIV-1 viral strains. rAAV9-mediated IgG expression varied between bNAb clones and mouse strains, with C57BL/6 mice exhibiting higher bNAb titers following rAAV delivery. Although CombiMab2 treatment elicited a higher IgG titer than CombiMab1, both combinations resulted in neutralization of all the viral strains from the global HIV-1 panel. Our data highlight the potential of AAV vectors as a long-term option for HIV-1 therapy.

## 1. Introduction

Human immunodeficiency virus (HIV-1) infection, and the subsequent progression to the acquired human immunodeficiency syndrome (AIDS), remains incurable and is one of the more pressing issues in healthcare. According to the WHO 2022 data, there are 39 million people living with HIV-1 [1]. HIV-1 infection is treated with antiretroviral therapy (ART), which suppresses the viral load and transforms HIV-1 into a manageable but incurable lifelong illness. However, long-term use of ART is associated with a host of side effects that reduces patient adherence to treatment and, as a consequence, promotes viral resistance to ART [2]. Thus, it is crucial to explore new avenues for the treatment of HIV-1.

The use of broadly neutralizing antibodies (bNAbs) capable of neutralizing HIV-1 viral strains may be a potential new approach to therapy. The first HIV-1 human monoclonal antibodies with potent neutralization capacity were reported in the early 1990s, but the rapid identification and characterization of multiple bNAbs became possible after 2009 [3,4]. Since then, the pool of available bNAbs has grown significantly, with more than 200 different bNAbs being used against HIV-1, and the new generation of bNAbs exhibits improved virus neutralization potency and breadth [4,5]. As a result, bNAbs are being actively implemented into new treatment paradigms for preclinical and clinical testing. At least 10 bNAbs are currently in various stages of clinical trials, with some showing promising results by successfully reducing viral load following antibody-based treatment [6]. However, some of the major issues with the aforementioned passive monospecific bNAb transfer are the emergence of therapy resistant viral strains and the relatively short half-life of the circulating antibodies. HIV-1 drug resistance is an anticipated issue, as the virus is highly mutagenic, and so a combination of antiretroviral drugs that block different steps in the viral replication cycle is used to combat this. An analogous approach with antibody therapy would be to use a mix of bNAbs targeting different viral envelope epitopes. Several in vitro studies have demonstrated that bNAb combinations have improved efficacy and breadth of neutralization, providing the most cellular protection against the virus [7,8].

The short half-life of passively transferred antibodies requires frequent repeated injections of bNAbs, which, given the need for lifelong therapy, makes this treatment paradigm complicated and expensive. Additionally, a gradual drop in anti-HIV antibody concentration promotes the development of drug-resistant viral strains. A promising approach to facilitating antibody persistence in the body is to deliver DNA sequence-encoding antibodies through an AAV vector, which can ensure stable long-term expression of antibodies [9,10]. This treatment framework could potentially improve therapy adherence, lower the cost of treatment, and reduce the dependance on ART, thereby alleviating some of its associated toxicity and enhancing the quality of life for people living with HIV-1.

Here, we used a mouse model to investigate the dynamics of in vivo anti-HIV bNAbs expression following the administration of three AAV vectors that encode different antibodies. In addition, we assessed the anti-HIV efficiency of treatment with various bNAb combinations by quantifying the neutralizing activity of mouse sera against a global panel of HIV-1 pseudoviruses.

## 2. Materials and Methods

### 2.1. Cell Lines

HEK293FT cells (Invitrogen, Carlsbad, CA, USA) were used to produce broadly neutralizing antibodies, HIV-1 pseudoviral particles and rAAV. Cells were cultured at 37 °C and 5% CO_2_ in DMEM (Gibco, Langley, OK, USA) and supplemented with 10% FBS (Gibco, Waltham, MA, USA), 10mM HEPES (Gibco, Waltham, MA, USA) and 0.1 mM nonessential amino acids (Gibco, Waltham, MA, USA).

To assess the efficiency of the neutralization of HIV-1 pseudoviral particles, a TZM-bl cell line (NIH AIDS Reagent Program, Manassas, VA, USA) was used. TZM-bl cells were cultured at 37 °C and 5% CO_2_ in complete DMEM (cDMEM), i.e., DMEM supplemented with 10% FBS and 25 mM HEPES (Gibco, Langley, OK, USA).

### 2.2. Plasmid Construction

Plasmid vector pAAV-GFP (VPK-411-SER6, Cell Biolabs, San Diego, CA, USA), containing AAV2 ITR (Inverted Terminal Repeat) were used for antibody vector construction. For this, the functional vector elements were inserted between the two ITRs in the following order: CMV_EF1α promoter, IgG-encoding segment (heavy and light chains connected by a GT2A peptide) and 3′-regulatory region of the transgene (Supplementary Figure S1). The cloning procedure and vector-related sequences (Supplementary Tables S1–S4) are provided in the Supplementary Materials section.

### 2.3. AAV Vector Preparation and Purification

HEK-293FT cells were seeded into two-level CellStack culture vials with an area of 1272 cm^2^ (Corning, Corning, NY, USA). The following day, cells were transfected with three plasmids: pAAV-Helper (Cell Biolabs, San Diego, CA, USA), pAAV-RC-9 plasmid (also from Cell Biolabs), encoding proteins of the 9 serotype capsid and the antibody-encoding vector plasmid. Polyethylenimine hydrochloride (PEI MAX, MW 40,000; from Polyscience Inc., Warrington, PA, USA) was used as a transfection agent. After 24 h, the growth medium was replaced with a serum-free OptiMem Reduced Serum Medium (Gibco) and the cells were incubated at 37 °C and 5% CO_2_ for 48 h. HEK-293FT cells were then harvested from the vials, precipitated by centrifugation, and resuspended in 6 mL of lysis buffer (containing 50 mM Tris-HCl, pH 8.0, 150 mM NaCl, and 2 mM MgCl_2_). The suspension underwent three freeze–thaw cycles followed by Benzonase (Merck, Darmstadt, Germany) treatment at 37 °C for 30 min and being clarified by centrifugation. The resulting supernatant was filtered through a 0.45 μm pore size filter membrane (TPP, Trasadingen, Switzerland).

Purification of rAAV was carried out using ultracentrifugation in the iodixanol gradient, based on a previously described protocol [11], which we optimized using Visipak^®^ (GE Healthcare, Chicago, IL, USA) [12]. Samples of the AAV vectors obtained after ultracentrifugation were dialyzed against phosphate buffered saline (PBS, Boston, MA, USA) with 0.001% Pluronic F-68 (Gibco) and concentrated using 100 kDa Amicon Ultra-15 concentrators (Merck). Aliquots of 50–100 μL were prepared and stored at −80 °C.

### 2.4. rAAV Quantification

The number of viral genomes per milliliter (vg/mL) was measured using droplet digital PCR (ddPCR) with the primers Fw: GGAACCCCTAGTGATGGAGTT, Rv: CGGCCTCAGTGAGCGA and the probe FAM-CACTCCCTCTCTGCGCGCTCG-BHQ1, which were complementary to the ITR sequences of AAV [13]. To achieve this, we isolated the total DNA from the samples using the “AmpliTest Ribo-prep” reagent kit (FSBI CSP FMBA, Moscow, Russia), according to the manufacturer’s instructions. ddPCR was performed using an automated sample loading system in a QX200™ AutoDG™ droplet generator (Bio-Rad, Hercules, CA, USA) and a C1000 Touch thermal cycler amplifier (Bio-Rad).

### 2.5. Antibody Expression in HEK293FT Cells Following Transduction with AAV Vectors

HEK293FT cells were seeded into a 6-well plate at a density of 1.2 × 10^5^ cells/cm^2^ and transduced with an AAV vector at a multiplicity of infection (MOI) of 1 × 10^6^ or 3 × 10^5^ genomes per cell. After 72 h, the cell culture medium was harvested to quantify antibody production.

### 2.6. Mice

C57BL6 or F1 CBA x C57BL hybrid female mice aged 6–8 weeks and weighing 19–23 g were used in the experiments. For the single-site treatment arm, 100 μL of the AAV vector combination was injected into to the femoral muscle. For the multiple-site treatment arm, each unique AAV vector solution was injected to a distinct site, 100 μL into the left thigh and 100 μL into the right thigh, and the remaining vector solution was split into 50 μL for each forelimb. Blood sampling was performed from the femoral vein in a volume of 50–100 μL. At the end of the experiment, the animals were humanely euthanized and blood was collected via cardiac puncture. The blood samples were incubated at room temperature for 30 min and then centrifuged at 2500× *g* for 25 min to isolate the serum. Mouse sera was then heat inactivated at 56 °C for 30 min.

To measure the concentration of antibodies, a Human IgG ELISA Antibody Pair Kit (StemCell, Vancouver, BC, Canada) was used. The procedure was carried out according to the manufacturer’s instructions.

### 2.7. Production of HIV-1 Pseudoviruses

To obtain HIV-1 pseudoviral particles, HEK293FT cells were seeded in a 75 cm^2^ culture flask and co-transfected with a pSG3DENV vector (6.9 μg) and a plasmid encoding the corresponding Env protein from the global HIV-1 panel (3.5 μg) [14]. A panel of Global HIV-1 Env Clones (cat# 12670) was obtained through the NIH AIDS Reagent Program, Division of AIDS, NIAID, NIH, from Dr. David Montefiori. PEI MAX (Polysciences Inc., Warrington, PA, USA) was used for transfection. The DNA:PEI ratio was 1:3. Transfection and collection of the cell supernatant containing pseudoviral particles was performed according to a described protocol [15]. A viral supernatant was dialyzed against phosphate buffered saline (PBS) and concentrated using 100 kDa Amicon Ultra-15 concentrators (Merck Millipore, Darmstadt, Germany). The concentrated pseudoviral particles were aliquoted and stored at −80 °C.

### 2.8. Titration of HIV-1 Pseudoviruses

Titration of pseudoviruses was carried out on TZM-bl cells, which contain the HIVsensitive luciferase transgene, according to the protocol described by Sarzotti-Kelsoe [16]. Viral infection was carried out in a 225 μL reaction. A concentrated pseudovirus was serially diluted five-fold in 125 μL of cDMEM in 96-well plate. Then, a 100 μL suspension containing 10,000 TZM-bl cells in cDMEM with 8 μg/mL polybrene (Sigma–Aldrich, St. Louis, MO, USA) was added to each well. All samples were assayed in triplicate. Twenty-four hours after transduction, the medium was changed to cDMEM. Seventy-two hours after transduction, the luciferase activity was estimated using a Bright-Glo reagent (Promega, Madison, WI, USA) according to the manufacturer’s instructions. The luminescence level was measured on a Tecan Infinite 200 Pro luminometer (Tecan Group Ltd., Männedorf, Switzerland). Then, an RLU (relative luminescence unit) versus virus dilution curve was plotted, and the volume required to achieve a luminescence of 200,000 RLU was determined. This volume was used in the following neutralization assays.

### 2.9. Neutralization Assay

The neutralization reaction was carried out according to the previously described protocol [17]. Briefly, five-fold dilutions of the mouse serum were mixed with the previously defined volume of pseudovirus stock in a 96-well plate, and cDMEM was added up to a volume of 125 μL. Samples were incubated for 1.5 h at 37 °C. Then, a 100 μL suspension containing 10,000 TZM-bl cells in cDMEM with 8 μg/mL polybrene (Sigma–Aldrich, St. Louis, MO, USA) was added to each well. Twenty-four hours after transduction, the medium was changed to cDMEM, and, 72 h after transduction, the RLU was obtained as described above.

The calculation of IC50 and IC80 was carried out in the Excel 2019 (Microsoft Corporation, Redmond, WA, USA) program according to the guidance provided by the Montefiori DC laboratory [17]. RLU values derived from the analyzed wells were normalized to the RLU of the control samples, which contained the same pseudovirus and the same dilution of serum from control mice not treated with an AAV. The obtained value was converted to the neutralization percentage. Then, the neutralization percentage was plotted against the concentration of antibodies on a logarithmic scale, and a nonlinear regression model was built. IC50 and IC80 values were defined as the antibody concentration needed to achieve a 50% and 80% pseudoviral neutralization, respectively. For the IC50 and IC80 calculations, we used bNAb concentrations in pooled sera measured by an ELISA.

### 2.10. Statistical Analyses

Statistical analyses were performed using Prism 6.0 (GraphPad Software). The mean and standard deviation were determined. Nonparametric data were analyzed by a two-tailed Mann–Whitney U test (for two groups). *p* values of <0.05 were considered significant (*, *p* < 0.05; **, *p* < 0.01).

## 3. Results

### 3.1. N6, 10E8, and 10-1074 Expression in HEK293FT Cells Following rAAV Transduction

**Figure 1:**
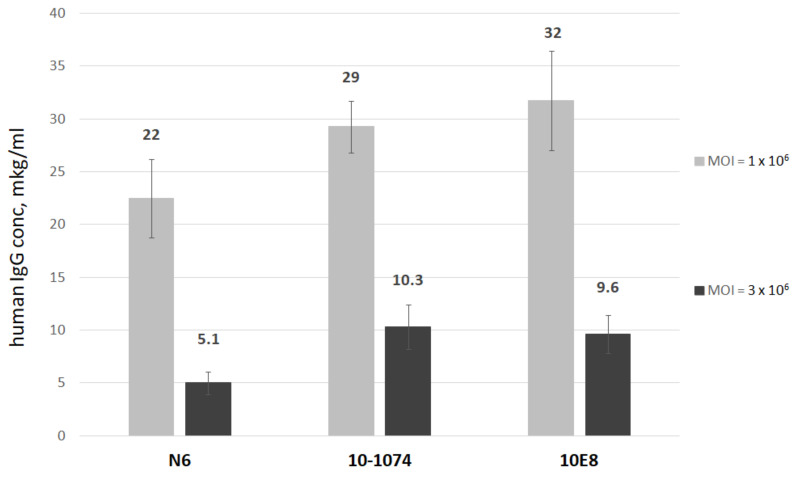
N6, 10E8 and 10-1074 bNAb expression in HEK293FT after rAAV transduction. Antibody concentrations in the culture media following the transduction of HEK293FT cells with AAV vectors encoding bNAbs. Culture media was collected 72 h after transduction, and human IgG concentration was quantified by an ELISA. Data are presented as means ± SEM from two independent experiments.

For an rAAV-based combination bNAb therapy against HIV-1, a combination of N6, 10E8 and 10-1074 antibodies targeting different viral envelope epitopes was selected, as this mix effectively neutralized a wide range of HIV-1 strains [8]. N6 targets the CD4-binding site (CD4bs) of the HIV-1 envelope [18], 10E8 targets the membrane-proximal external region of gp41 [19] and 10-1074 targets the V3 envelope region [20]. Using serotype 9 AAV vectors, we synthesized rAAV-N6, rAAV-10E8 and rAAV-10-1074, encoding N6, 10E8 and 10-1074 antibodies, respectively. The resulting vectors were then used to transduce HEK293FT cells with a MOI of 3 × 10^5^ or 3 × 10^6^ vg per cell. As shown in Figure 1, the rAAV-infected cells successfully expressed and secreted antibodies into the culture fluid. The antibody expression levels were positively correlated to the MOI.

### 3.2. N6, 10E8, and 10-1074 Expression in CBAxC57Bl Mice

**Figure 2:**
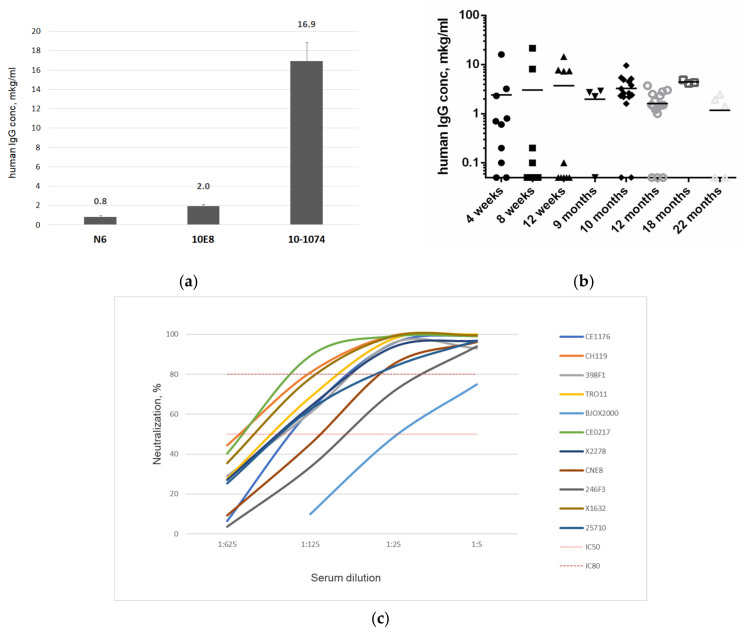
N6, 10E8, 10-1074 bNAbs and CombiMab1 expression and activity in CBAxC57Bl mice. (**a**) Antibody concentration in pooled sera from CBAxC57Bl mice injected with rAAV encoding N6, 10E8 or 10-1074 after 12 weeks. (**b**) The dynamics of human IgG expression in mouse sera up to 22 months after CombiMab1 delivery. (**c**) Neutralization ability of pooled sera from CBAxC57Bl mice treated with CombiMab1 against the global panel of HIV-1 pseudoviruses.

We first ran a pilot study to show that each AAV vector used is capable of maintaining an in vivo bNAb expression. For this, we set up three CBAxC57Bl mouse groups, receiving 2 × 10^11^ vg of an rAAV encoding N6, 10E8 or 10-1074 antibody. After 12 weeks, the mouse serum was collected. The mouse sera were then pooled according to the experimental group, and human IgG concentration was quantified by an ELISA (Figure 2a). The introduction of different antibody constructs led to varying serum concentrations of antibodies, which fundamentally differed from the results obtained in the in vitro experiments (Figure 1). The 10-1074 antibody had the highest-level in vivo expression.

We then investigated the dynamics of antibody expression after administering a combination of AAV vectors. A 1:1:1 mix of rAAV-N6, rAAV-10E8 and rAAV-10-1074 (hereinafter referred to as CombiMab1) was administered to CBAxC57Bl mice at a cumulative dose of 6 × 10^11^ vg per animal. The concentration of human IgG in mouse sera was measured at various time points (from 4 weeks to 22 months) following CombiMab1 delivery (Figure 2b). The average concentration of human antibodies in mouse sera ranged from 1.2 to 4.4 μg/mL at various time points. At weeks 4, 8 and 12, the antibody expression became quite heterogeneous, with notable differences in IgG expression between individual animals. In some cases, the serum human IgG concentration dropped below detection levels. Human antibodies were still detectable in mouse sera at 22 months following rAAV treatment.

### 3.3. Neutralization Potency of Mouse Sera against the Global Panel of HIV-1 Pseudoviruses

The neutralization ability of mouse sera was tested against 11 psedoviruses from the global panel of HIV-1 Env reference strains [14]. For the neutralization reaction, mouse sera with high antibody titers (more than 1 μg/mL) obtained at weeks 4, 8 and 12 were pooled to a final concentration of 9.4 μg/mL. For each pseudovirus, the neutralization reaction was carried out with a series of five-fold dilutions of pooled mouse sera. Curves illustrating the relationship between the effectiveness of neutralization to serum dilution for the panel of pseudoviruses are shown in Figure 2c.

The pooled sera at a 1:5 dilution neutralized all of the pseudovirus strains with a >80% efficiency, with the exception of BJXO2000, which was neutralized with a 75% efficiency. At a 1:25 dilution, most viruses were neutralized with a >80% efficiency, except for BJXO2000 and 246F3 with 48% and 71%, respectively. Thus, CombiMab1 delivery to CBAxC57Bl mice led to a long-term persistence of antibodies in blood which provided a neutralizing activity of the mouse sera against a global panel of HIV-1 Env reference strains. However, the cumulative concentration of human IgGs was quite low, potentially owing to the low level of N6 and 10E8 in vivo expression. In addition, antibody expression in the first 3 months following CombiMab1 administration was quite variable. Heterogenous antibody expression may be linked to the use of a hybrid CBAxC57Bl mouse line, and, as such, we conducted further studies using inbred mice.

### 3.4. bNAb Expression in C57BL/6 Mice

**Figure 3:**
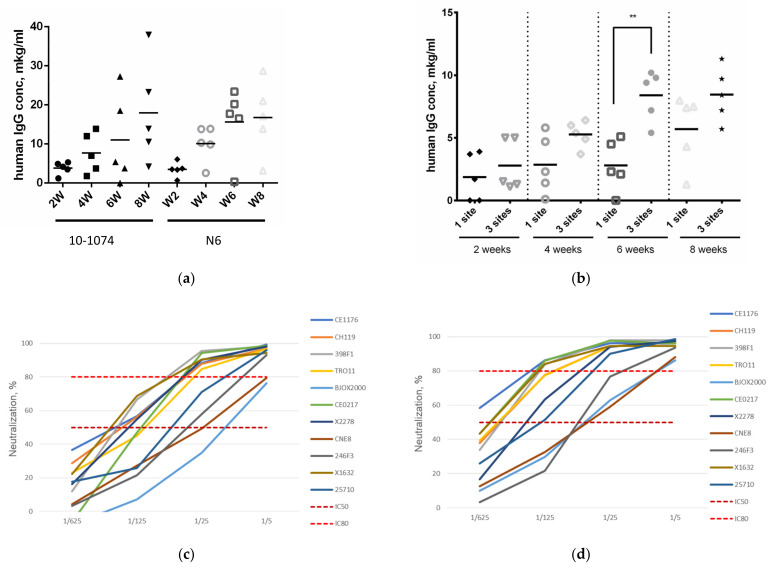
N6, 10-1074 bNAbs and CombiMab1 expression and activity in C57Bl/6 mice. (**a**) Human IgG concentration in C57BL/6 mouse serum at 2, 4, 6, or 8 weeks post AAV vector delivery, encoding 10-1076 or N6 antibodies. The mean values for each group are represented by the bar. (**b**) Human IgG concentration in C57BL/6 mouse serum at 2, 4, 6, or 8 weeks post CombiMab1 administration, either at one site or three different sites. ** *p* < 0.01 (0.0079) (**c**,**d**) Neutralization ability of pooled sera from C57Bl/6 mice who received CombiMab1 at one injection site (**c**) or multiple injection sites (**d**).

The first step in the study of antibody expression in an inbred mouse line was the assessment of N6 and 10-1074 antibody expression in C57BL/6 mice. Each animal received an intramuscular injection of an AAV vector encoding the N6 or the 10-1074 antibody at a dose of 2 × 10^11^ vg. Human IgG concentrations were then quantified in mouse sera at various time points (Figure 3a). Over time, the antibody titers rose, reaching an average of 15–18 μg/mL at 6–8 weeks. Contrary to the CBAxC57Bl mice, which had low titers of the N6 antibody (Figure 2a), the inbred C57BL/6 mice expressed the N6 antibody at levels similar to 10-1074.

Next, we administered CombiMab1 to C57BL/6 mice following the same protocol as with the hybrid mouse line. We additionally explored two different routes of CombiMab1 delivery. The first group of animals was injected with a mixture of rAAV-N6, rAAV-10-1074 and rAAV-10E8 into the femoral muscle at a dose of 6 × 10^11^ vg (2 × 10^11^ vg of each vector). The second group of animals received the same dosing of CombiMab1, except each vector was administered to a different region: 100 μL of rAAV-N6 to the left thigh, 100 μL of rAAV-10-1074 to the right thigh and 50 μL of rAAV-10E8 to each of the two front shoulders. Vector injection into different sites may contribute to an increase in antibody expression by expanding the area of vector delivery, thus increasing the number of transduced cells. Administering different constructs at separate injection sites may also reduce the formation of chimeric antibodies, which can occur when different AAV vectors enter the same cell. After 2, 4, 6, and 8 weeks, human IgG concentration in the mouse-sera was quantified (Figure 3b).

Over the course of 8 weeks, both groups exhibited a steady rise in antibody titers. On average, animals with multiple sites of vector delivery had greater antibody titers than animals from the other group at each investigated point. At week 6, the difference was significant (*p* < 0.05 Mann–Whitney test).

### 3.5. Neutralization Potency of CombiMab1-Treated C57BL/6 Mouse Sera against the Global Panel of HIV-1 Pseudoviruses 

We assessed the neutralization capacity of the sera obtained from C57BL/6 mice treated with CombiMab1 with varying routes of vector delivery. For the neutralization reaction, sera from mice in each group were pooled; the multiple injection site group had a pooled antibody titer of 9.5 µg/mL and the one site group had 4.0 μg/mL. A global HIV-1 panel was used to determine the neutralizing activity of the pooled sera, and the results are shown in Figure 3c,d. The BJOX20000 and CNE8 viral strains had the poorest neutralization outcomes. For these viruses, the neutralization efficiency of the 1:5 diluted pooled sera from the one site group was above 50% but did not reach 80%. For the remainder of the viral strains, the 1:5 serum dilution for both groups had >80% neutralization efficiency.

Pooled sera obtained from mice receiving CombiMab1 at multiple injection sites had better neutralization outcomes, with the 1:5 dilution neutralizing all viral strains with a >80% efficiency. Similar to the one injection site group, the neutralization efficiency for BJOX20000 and CNE8 viral strains was lower than for the other pseudoviruses.

To compare the neutralization potency of the sera from the two treatment groups without the influence of their respective IgG concentrations, we computed the IC50 and IC80 values (Supplementary Table S5). No significant differences in neutralization efficiency were found between the sera from the two experimental groups.

### 3.6. Expression of VRC07-523, 10-1074 and PGDM1400 Antibodies and Their Combination in C57BL/6 Mice 

Given the low expression of the two out of the three antibodies making up CombiMab1, in the initial experiments (Figure 2a) we assembled another bNAb combination. We found that 10-1074 was retained due to its consistently high expression among different mouse lines. N6 was replaced by VRC07-523, which also binds the CD4-binding site of the HIV-1 envelope [21], and 10E8 was replaced by PGDM1400, which targets the V1/V2 epitope [22]. The in vitro neutralization potency of VRC07-523 and PGDM1400 was validated in our previous study [8]. AAV vectors encoding VRC07-523 and PGDM1400 were designed and produced. The rAAV combination consisting of a 1:1:1 mix of AAV vectors encoding VRC07-523, PGDM1400 and 10-1074 antibodies was denoted as CombiMab2.

**Figure 4:**
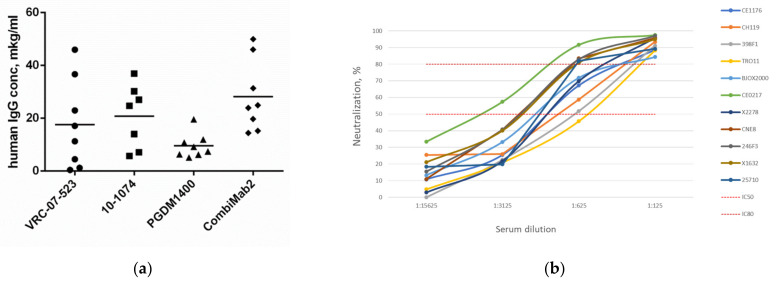
VRC-07-523, 10-1074, PGDM1400 and CombiMab1 expression and activity in C57Bl/6 mice. (**a**) Serum IgG concentration (μg/mL) in C57BL/6 mice 4 weeks after rAAV administration. The mean is indicated by the dash (n = 8); (**b**) neutralization efficiency of pooled sera from C57BL/6 mice, collected 4 weeks after treatment with CombiMab2, against 11 strains of the global panel pseudoviruses.

C57BL/6 mice were intramuscularly injected with either AAV vectors encoding individual antibodies or CombiMab2. The vectors encoding individual antibodies were administered at a dose of 2 × 10^11^ vg per mouse, while CombiMab2 was administered at 6 × 10^11^ vg (2 × 10^11^ vg of each vector). The serum IgG concentration after 4 weeks is shown in Figure 4a.

VRC07-523 and 10-1074 constructs were expressed at a similar level at 17.5 and 20.8 μg/mL, respectively. PGDM1400 had a lower expression at 9.6 μg/mL, but this difference was not statistically significant. CombiMab2 had an average IgG expression of 28 μg/mL.

When comparing the C57BL/6 mouse serum IgG concentration of the two rAAV combinations, CombiMab2 had a higher antibody titer at 28 μg/mL versus CombiMab1 at 4.1 μg/mL.

### 3.7. Neutralization Potency of CombiMab2-Treated C57BL/6 Mouse Sera against the Global Panel of HIV-1 Pseudoviruses 

To assess the neutralization efficacy of CombiMab2, sera from all animals receiving CombiMab2 was pooled and tested against a global panel of HIV-1 pseudoviruses. The resulting data are presented in Figure 4b. The pooled serum demonstrated a high neutralizing ability, and all viruses from the global panel were neutralized with a >80% efficiency at a 1:125 serum dilution. At the 1:625 serum dilution, five viruses were neutralized with a >80% efficiency, four with a >50% efficiency, and one with a near 50% efficiency.

For the purpose of comparing the neutralization capability of sera obtained from mice treated with CombiMab1 versus CombiMab2 independent of serum IgG concentration, we computed IC50 and IC80 values for each viral strain (Table 1). For CombiMab2, IC50 did not surpass 0.03 μg/mL and IC80—0.16 μg/mL. For the viruses BJOX2000, CE0217, CNE8, 246F3 and 25710, the neutralization efficiencies of sera obtained from animals treated with CombiMab2 significantly exceeded those of CombiMab1.

## 4. Discussion

The use of broadly neutralizing antibodies against HIV-1 is a promising approach to HIV-1 therapy, as bNAbs have demonstrated their efficacy in multiple preclinical and clinical studies [6]. However, some of the major challenges of passive bNAb transfer are the short half-life of antibodies and the rapid development of viral resistance. In this work, we investigated two combinations of AAV vectors each encoding three different bNAbs, denoted as CombiMab1 and CombiMab2, which could form the basis of an HIV-1 gene therapy.

To determine the optimal AAV vector delivery system, we conducted an in vivo pilot experiment comparing 10-1074 antibody expression delivered by an AAV vector of 8, 9 or DJ serotypes to CBAxC57Bl mice. Administration of AAV9-10-1074 led to the highest IgG expression, while AAV8-10-1074 resulted in undetectable IgG levels (Supplementary Figure S2). Based on these data, the AAV9 vector was selected for our further experiments. However, our data differed from that of other animal studies that reported AAV8 as being effective at mediating antibody expression in mice [23,24,25]. On the other hand, clinical studies using AAV1 or AAV8 for anti-HIV-1 bNAbs expression have shown either low or undetectable antibody titers [26,27].

To assess AAV vector infection ability and antibody production, we transduced HEK293FT cells with AAV9 vectors encoding various anti-HIV-1 antibody clones. The cells achieved a stable expression of the selected antibodies with little variation in expression. Moving to in vivo antibody expression in CBAxC57Bl hybrid mice, we saw antibody-dependent differences in expression. This is expected, as in vivo conditions vary significantly from the in vitro model, especially with regards to a potential immune response to both the vector and transgene product. But in C57BL/6 mice expression of N6 and 10-1074 did not differ. Furthermore, C57BL/6 animals receiving CombiMab1 had higher human IgG titers (median 4.8 μg/mL) in comparison to their CBAxC57Bl counterparts (median 0.75 μg/mL) in the same treatment group (Supplementary Figure S3). The effect of animal strain on expression levels was also shown by Balazs et al. (2011) [23], but they reported more moderate differences. It may be that different strains of mice may have distinct immune responses to both rAAV injection and secretion of a foreign protein.

Individual differences in animal responses to rAAV injection were also observed. In CBAxC57Bl mice, treatment with CombiMab1 yielded heterogeneous IgG expression. Some animals showed a decrease in IgG titers below detection levels, whereas others maintained stable expressions ranging from 1.3 to 4.9 μg/mL over 22 months. As expected, the inbred C57BL/6 mice showed less internal variation. High variability of antibody titers within a group, with more than a 100-fold difference in concentration, was also reported by van denBerg et al. (2019) [24] despite the use of inbred female mice. The authors ascertained that, in many cases, the low human IgG titer was associated with the production of anti-antibodies. So, an immune response to the transgene product may be the frequent cause for the IgG titer variability. However, the immune response did not account for all cases of low transgene expression in the van den Berg study. 

Despite heterogeneity in antibody expression among the animals, bNAb expression lasted for as long as 22 months post CombiMab1 injection in CBAxC57Bl hybrid mice. These timelines are consistent with other studies, wherein rAAVs supported in vivo expression for 6–12 months following intramuscular vector delivery [28,29]. These data highlight the potential of AAV vectors as a long-term treatment option.

We then evaluated the efficiency of CombiMab1 through neutralization reactions against a panel of HIV-1 pseudoviruses. Pooled sera from each CBAxC57Bl mice and C57BL/6 inbred mouse line was active against all pseudoviruses from the global HIV-1 panel. However, neutralization of BJXO2000, 246F3 and CNE8 pseudoviruses was less effective than the neutralization of other pseudoviruses (Figure 2c and Figure 3d).

Within our experiments, we tested two options for administering the combination therapy. In the first case, CombiMab1 was administered as a mixture of three AAV vectors into the femoral muscle. In the second case, each of the three AAV vectors was injected separately into different muscles. With multiple-site injections, a significantly higher serum IgG concentration was achieved. Similar results were observed in a study by Welles et al. (2018) in rhesus monkeys, wherein the AAV8 vector encoding monoclonal antibodies against the simian immunodeficiency virus was injected into one or more sites [30]. According to that study, splitting the vector dose among several sites led to an increase in human IgG concentration. The authors reasoned that, in the case of a single intramuscular injection, the number of muscle cells available for transduction is limited by physical parameters, so dose distribution across multiple sites increases the number of cells transduced and thereby increases antibody expression. A similar conclusion was reached by a clinical study of AAV8-VRC07 vector in which several injection sites led to an increase in VRC07 bNAb [27].

With regards to neutralization efficiency, pooled serum from animals receiving multiple site vector injections was more effective than the sera from the single injection site group. This could be due to differences in antibody concentrations between groups but also due to non-functional antibody formation when rAAVs encoding different antibodies enter the same cell and promote a cross-assembly of bNAb constructs. As there were no significant differences between the IC50 and IC80 for the two different routes of vector delivery (Supplementary Table S5), we concluded that the higher neutralization ability of the multiple site group was due to a higher serum IgG concentration. 

The experiments examining the second combination of vectors (CombiMab2), encoding VRC07-523, PGDM1400 and 10-1074 antibodies, demonstrated the significant advantages of this combination. CombiMab2 showed superiority over CombiMab1 in terms of both expression, having a seven-fold higher serum IgG and neutralization efficiency. Sera from the CombiMab2 group exhibited significantly lower IC50 and IC80 values for 5 of the 11 pseudoviruses compared to the CombiMab1 group (Table 1). The importance of selecting an optimal combination was shown both in our previous work, where we compared the in vitro neutralization breadth and potency of various bNAb clones and their combinations, and in the in vivo study by Yao et al. (2023) [25]. In the latter study, AAV8-vectors encoding different anti-HIV bNAbs were administered in either dual or quadruple combinations to mice [25]. Interestingly, the blood IgG concentration after double mix (PG16 and 10E8 antibodies) administration was higher compared to the four antibody combination (PG9, PG16, 10E8 and NIH45-46) [25]. Moreover, serum obtained from the double combination group had a greater breadth and higher neutralization capacity against a panel of pseudoviruses than the four antibody group. Potentially, this may have happened due to a mutual inhibition of antibodies in the quadruple mix or a difference in IgG expression. Therefore, it is important to optimally select the combination of antibodies to include in a therapeutic treatment.

## 5. Conclusions

The use of an optimally selected combination of rAAVs encoding different bNAbs is a promising strategy for the treatment of HIV, which can increase the effectiveness of passive immunization and protect the body from a wide range of viral strains.

## Figures and Tables

**Table 1:** IC50 and IC80 values for pooled sera obtained from C57BL/6 mice treated with either CombiMab1 or CombiMab 2.

	IC50 (μg/mL)		IC80 (μg/mL)	
	CombiMab2	CombiMab1	CombiMab2	CombiMab1
CE1176	0.019	0.017	0.106	0.143
CH119	0.016	0.020	0.101	0.162
398F1	0.027	0.030	0.131	0.127
TRO11	0.030	0.031	0.162	0.207
BJOX2000	0.017	0.245 *	0.106	1.274 *
CE0217	0.005	0.051*	0.034	0.171 *
X2278	0.019	0.032	0.082	0.162
CNE8	0.012	0.136 *	0.055	0.947 *
246F3	0.011	0.098 *	0.054	0.459 *
X1632	0.010	0.020	0.059	0.077
25710	0.015	0.048 *	0.068	0.330 *

* The IC50 and IC80 values for CombiMab1 that are three-fold or greater than the corresponding values for CombiMab2.

## Data Availability

The original contributions presented in the study are included in the article and Supplementary Materials, further inquiries can be directed to the corresponding author.

## Supplementary Materials

The following supporting information can be downloaded at: https://www.mdpi.com/article/10.3390/v16081296/s1. Supplementary data: cloning procedure and vector-related sequences; Figure S1: schematic representation of pAAVcore and pAAV expression plasmids Figure S2: human antibody concentration in sera from CBAxC57Bl mice 12 weeks after administering AAV vectors of 8, 9, or DJ serotypes; Figure S3: human antibody concentration in sera from CBAxC57Bl or C57Black mice 4 weeks after administering of Combimab1; Table S1: sequences of regulatory elements in pAAVcore plasmid; Table S2: nucleotide sequences encoding the furin cleavage site and HGH signal peptides for heavy and light chain; Table S3: protein sequences of antibody light chains; Table S4: protein sequences of antibody heavy chains; Table S5: IC50 and IC80 values for pooled sera obtained from C57BL/6 mice injected with Combimab1 either at one site or at three different sites.
