# Supplementary material for: Triple Combinations of AAV9-Vectors Encoding Anti-HIV bNAbs Provide Long-Term In Vivo Expression of Human IgG Effectively Neutralizing Pseudoviruses from HIV-1 Global Panel
Source: Viruses. 2024 Aug 14;16(8):1296. doi: 10.3390/v16081296 (PMC11359378; doi:10.3390/v16081296)
Supplement: Supplementary file 1 [file viruses-16-01296-s001.zip › viruses-3128006-supplementary.pdf]

Supplementary

Cloning procedure and vector-related sequences

Plasmid construction

To obtain plasmid pAAVcore (Figure S1a) we synthesized two DNA fragments. First fragment encoded CMV_EF1α hybrid promoter flanked by NotI and ClaI/BamH1 restriction sites. Second fragment contained 3′-regulatory region flanked by BamH1/BglII and NotI. The sequences of CMV_EF1α and 3′-regulatory region are presented in Table S1. Two DNA fragments were inserted in pAAV-GFP plasmid (VPK-411-SER6, Cell Biolabs, USA) in NotI/BamH1 sites. As a result, we obtained pAAVcore plasmid (Figure S1a).

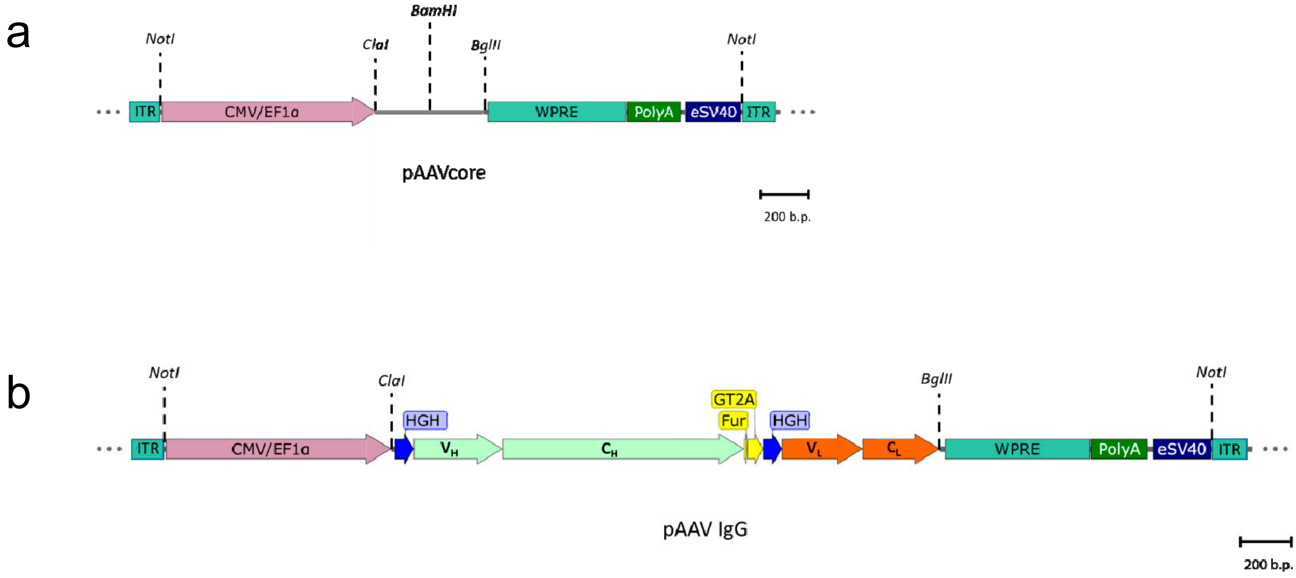

Supplementary Figure S1 (a) – Schematic representation of pAAVcore plasmid; (b) – Schematic representation of AAV expression plasmid; ITR – inverted terminal repeat, CMV/EF1a - hybrid promoter, WPRE - woodchuck hepatitis virus post-transcriptional regulatory element, PolyA – polyadenylation signal from simian virus 40 (SV40), eSV40 – SV40 enhancer sequence, HGH – human growth hormone signal peptide, V_H – heavy variable chain, C_H - heavy constant chain, V_L - light variable chain, C_L - light constant chain, Fur - furin cleavage site, GT2A - GT2A self-cleaving peptide.

Supplementary Table S1 Sequences of regulatory elements in pAAVcore plasmid

	Sequence 5′→3′
CMV_EF1α	*(NotI)gcggccgc*gctctggagacgcgttacataacttacggtaaatggcccgcctggctgaccgcccaacgacccccgcccattgacgtcaataatgacgtatgttcccatagtaacgccaatagggactttccattgacgtcaatgggtggagtatttacggtaaactgcccacttggcagtacatcaagtgtatcatatgccaagtacgccccctattgacgtcaatgacggtaaatggcccgcctggcattatgcccagtacatgaccttatgggactttcctacttggcagtacatctacgtattagtcatcgctattaccatgggtgaggctccggtgcccgtcagtgggcagagcgcacatcgcccacagtccccgagaagttggggggaggggtcggcaattgaaccggtgcctagagaaggtggcgcggggtaaactgggaaagtgatgtcgtgtactggctccgcctttttcccgagggtgggggagaaccgtatataagtgcagtagtcgccgtgaacgttctttttcgcaacgggtttgccgccagaacacaggtaagtccggcctccgcgccgggttttggcgcctcccgcgggcgcccccctcctcacggcgagcgctgccacgtcagacgaagggcgcaggagcgttcctgatccttccgcccggacgctcaggacagcggcccgctgctcataagactcggccttagaaccccagtatcagcagaaggacattttaggacgggacttgggtgactctagggcactggttttctttccagagagcggaacaggcgaggaaaagtagtcccttctcggcgattctgcggagggatctccgtggggcggtgaacgccgatgatgcctctactaaccatgttcatgttttctttttttttctacaggtcctgggtgacgaacagc*atcgat(ClaI)*
3′-regulatory region: **Woodchuck Posttranscriptional Regulatory Element (WPRE)**, polyadenylation signal (PolyA), **eSV40 enhancer sequence**	*(BglI)agatct***ataatcaacctctggattacaaaatttgtgaaagattgactggtattcttaactatgttgctccttttacgctatgtggatacgctgctttaatgcctttgtatcatgctattgcttcccgtatggctttcattttctcctccttgtataaatcctggttgctgtctctttatgaggagttgtggcccgttgtcaggcaacgtggcgtggtgtgcactgtgtttgctgacgcaacccccactggttggggcattgccaccacctgtcagctcctttccgggactttcgctttccccctccctattgccacggcggaactcatcgccgcctgccttgcccgctgctggacaggggctcggctgttgggcactgacaattccgtggtgttgtcggggaaatcatcgtcctttccttggctgctcgcctgtgttgccacctggattctgcgcgggacgtccttctgctacgtcccttcggccctcaatccagcggaccttccttcccgcggcctgctgccggctctgcggcctcttccgcgtcttcgccttcgccctcagacgagtcggatctccctttgggccgcctccccgcatc**ctcgaggctcgctgatcagcctcgactgtgccttctagttgccagccatctgttgtttgcccctcccccgtgccttccttgaccctggaaggtgccactcccactgtcctttcctaataaaatgaggaaattgcatcgcattgtctgagtaggtgtcattctattctggggggtggggtggggcaggacagcaagggggaggattgggaagacaatagcaggcatgctggggatctgagtgtcgataaggatctgaa**cgatggagcggagaatgggcggaactgggcggagttagggcgggatgggcggagttaggggcgggactatggttgctgactaattgagatgcatgctttgcatacttctgcctgctggggagcctggggactttccacacctggttgctgactaattgagatgcatgctttgcatacttctgcctgctggggagcctggggactttccacaccctaactgacacacattccacagc***gcggccgc(NotI)*

Next, we generated DNA coding sequences from amino acid sequences for each antibody. Antibody protein sequences are represented in Tables S3 and S4. To ensure secretion of heavy and light immunoglobulin chains both of them included HGH signal at the N-terminus. DNA coding sequences of HGH signals were different from each other in order to reduce

recombination events in the transgene (Table S2). Antibody-coding sequences were synthesized and inserted in pAAVcore using ClaI and BglII sites. Finally, we obtained plasmid vectors pAAV-VRC-07-523, pAAV-PGDM1400, pAAV-10-1074, pAAV-N6 and pAAV-10E8, which were used for AAV particle production. Schematic representation of the plasmid vectors is shown in Figure S1b.

Supplementary Table S2 Nucleotide sequences encoding the furin cleavage site and HGH signal peptides for heavy and light chain

	Sequence 5′→3′
**Coding sequence of furin cleavage site,** *coding sequence of GT2A*	**aggaagagaagg***ggatctggagagggcagaggaagtctgctgacatgtggtgatgtcgaggagaatcctggcccaatggctaccggcagcagaacaagcctgctgctggcttttggactgctctgtctcccctggttgcaagaaggctctgcc*
Coding sequence of heavy chain HGH	atggctactggctctcgaaccagcctcctgctggcatttgggttgctgtgtctgccatggctgcaggagggctctgcc
Coding sequence of light chain HGH	atggctaccggcagcagaacaagcctgctgctcgcttttggactgctctgtctcccctggttgcaagaaggcagcgcc

Supplementary Table S3 Protein sequences of antibody light chains

	Amino acid sequence
C_L of λ chain of human IgG1	QPKAAPSVTLFPPSSEELQANKATLVCLISDFYPGAVTVAWKADSS<br>PVKAGVETTTPSKQSNNKYAASSYLSLTPEQWKSHRSYSCQVTHE<br>GSTVEKTVAPTECS
C_L of κ chain of human IgG1	RTVAAPSVFIFPPSDEQLKSGTASVVCLLNNFYPREAKVQWKVDN<br>ALQSGNSQESVTEQDSKDSTYSLSSTLTLSKADYEKHKVYACEVT<br>HQGLSSPVTKSFNRGEC
C_H of human IgG1 (with an M428L and N434S substitutions)	ASTKGPSVFPLAPSSKSTSGGTAALGCLVKDYFPEPVTVSWNSGAL<br>TSGVHTFPAVLQSSGLYSLSSVVTVPSSSLGTQTYICNVNHKPSNT<br>KVDKKAEPKSCDKTHTCPPCPAPELLGGPSVFLFPPKPKDTLMISR<br>TPEVTCVVVDVSHEDPEVKFNWYVDGVEVHNAKTKPREEQYNST<br>YRVVSVLTVLHQDWLNGKEYKCKVSNKALPAPIEKTISKAKGQPR<br>EPQVYTLPPSRDELTKNQVSLTCLVKGFYPSDIAVEWESNGQPENN<br>YKTTPPVLDSDGSFFLYSKLTVDKSRWQQGNVFSCSV**L**HEALH**S**H<br>YTQKSLSLSPGK

Supplementary Table S4 Protein sequences of antibody heavy chains

	Amino acid sequence	Sourse
V_H of 10-1074	QVQLQESGPGLVKPSETLSVTCSVSGDSMNN YYWTWIRQSPGKGLEWIGYISDRESATYNPS LNSRVVISRDTSKNQLSLKLNSVTPADTAVY YCATARRGQRIYGVVSFGEFFYYYSMDVWG KGTTVTVSS	https://www.ncbi.nlm.nih.gov/protein/ PDB: 5T3X_H
V_L of 10-1074	SYVRPLSVALGETARISCGRQALGSRAVQWY QHRPGQAPILLIYNNQDRPSGIPERFSGTPDIN FGTRATLTISGVEAGDEADYYCHMWDSRSG FSWSFGGATRLTVLG	https://www.ncbi.nlm.nih.gov/protein/ PDB: 7UCG_K (a.a. № 1-109)
V_H of 10E8	EVQLVESGGGLVKPGGSLRLSCSASGFDFDN AWMTWVRQPPGKGLEWVGRITGPGEGWSV DYAAPVEGRFTISRLNSINFLYLEMNNLRME DSGLYFCARTGKYYDFWSGYPPGEEYFQDW GRGTLVTVSS	https://www.ncbi.nlm.nih.gov/protein/ PDB: 8SX3_H (a.a. № 1-131)
V_L of 10E8	SYELTQETGVSVALGRTVTITCRGDSLRSHY ASWYQKKPGQAPILLFYGKNNRPSGVPDRFS GSASGNRASLTISGAQAEDDAEYYCSSRDKS GSRLSVFGGGTKLTVLS	https://www.ncbi.nlm.nih.gov/protein/ PDB: 5JNY_B (a.a. № 1-110)
V_H of N6	RAHLVQSGTAMKKPGASVRVSCQTSGYTFT AHILFWFRQAPGRGLEWVGWIKPQYGAVNF GGGFRDRVTLTRDVYREIAYMDIRGLKPDDT AVYYCARDRSYGDSSWALDAWGQGTTVVV SA	https://www.ncbi.nlm.nih.gov/protein/ AOW41560.1

V_L of N6	YIHVTQSPSSLSVSIGDRVTINCQTSQGVGSDLHWYQHKPGRAPKLLIHHTSSVEDGVPSRFSGSGFHTSFNLTISDLQADDIATYYCQVLQFFGRGSRLHIK	https://www.ncbi.nlm.nih.gov/protein/ AOW41564.1
V_H of PGDM1400	QVHLTQSGPEVRKPGTSVKVSCKAPGNTLKTYDLHWVRSVPGQGLQWMGWISHEGDKKVIVERFKAKVTIDWDRSTNTAYLQLSGLTSGDTAVYYCAKGSKHRLRDYALYDDDGALNWAVDVDYLSNLEFWGQGTAVTVSS	https://www.ncbi.nlm.nih.gov/protein/ AIY25988.1
V_L of PGDM1400	DFVLTQSPHSLSVTPGESASISCKSSHSLIHGDRNNYLAWYVQKPGRSPQLLIYLASSRASGVPDRFSGSGSDKDFTLKISRVETEDVGTYYCMQGRESPWTFGQGTKVDIK	https://www.ncbi.nlm.nih.gov/protein/ AIY26001.1
V_H of VRC07-523	QVRLSQSGGQMKKPGDSMRISCRASGYEFINCPINWIRLAPGKRPEWMGWMKPRHGAVSYARQLQGRVTMTRDMYSETAFLELRSLTSDDTAVYFCTRGKYCTARDYYNWDFEHWGQGTPVTVSS	https://www.ncbi.nlm.nih.gov/protein/ PDB: 4OLW_H (a.a. № 1-125)
V_L of VRC07-523	SLTQSPGTLSLSPGETAIISCRTSQYGSLAWYQQRPGQAPRLVIYSGSTRAAGIPDRFSGSRWGPDYNLTISNLESGDFGVYYCQQYEFFGQGTKVQVDIKR	https://www.ncbi.nlm.nih.gov/protein/ AIM17881.1

Capsid Selection

The ability of different rAAV capsids to induce antibody expression *in vivo* was investigated. In the study, rAAVs with capsids 8, 9, and DJ encoding antibody 10-1074 were used. The rAAV vectors were administered intramuscularly to CBAxC57Bl mice in two doses: dose1 (2.0E+11 vector genomes per mouse) and dose 2 (5.0E+10 vector genomes per mouse), in a volume of 50 µl/mouse. Control animals received a control solution of 0.9% NaCl.

Twelve weeks after AAV administration, the concentration of antibodies in serum was measured using ELISA. The highest level of antibodies was achieved with the administration of rAAV-9.

Supplementary Figure S2

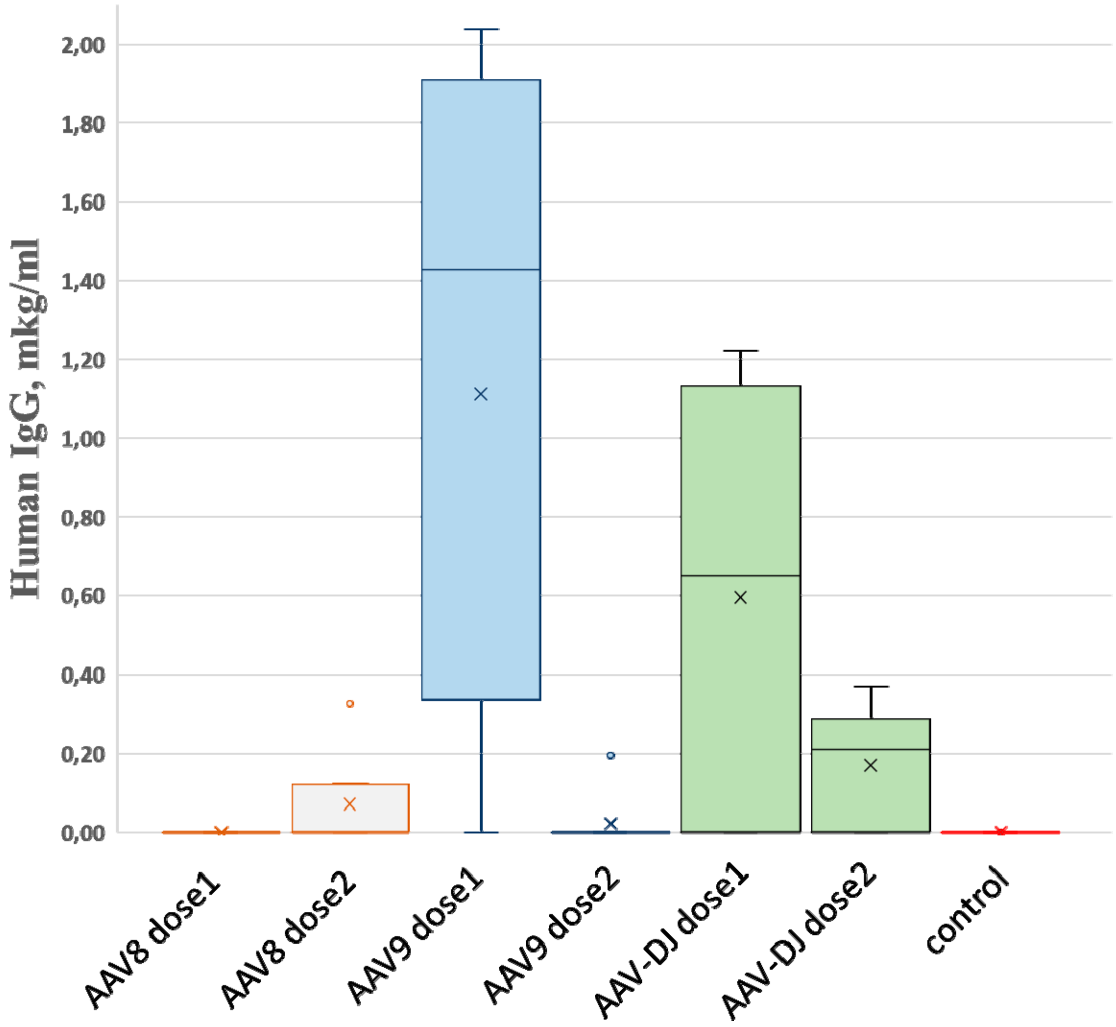

Figure S2 Human antibody concentration in sera from CBAxC57Bl mice 12 weeks after administering AAV vectors of 8, 9, or DJ serotypes. Dose 1 – $2*10^{11}$ vg per mouse, dose 2 – $5*10^{10}$ vg per mouse. The mean values are indicated by «×» symbol. The medians are depicted by line.

Supplementary Fig S3

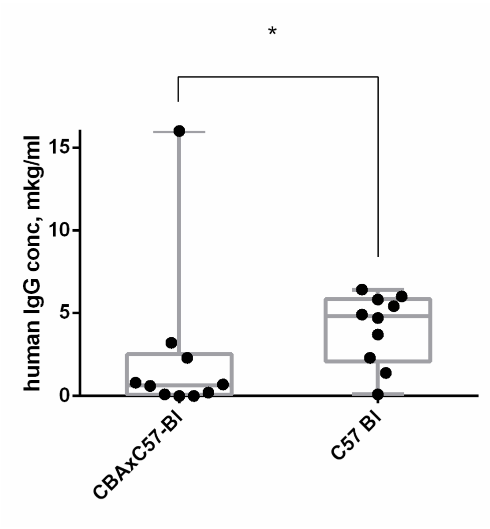

Figure S3 Human antibody concentration in sera from CBAxC57Bl or C57Black mice 4 weeks after administering of Combimab1. * p<0,05

Supplementary Table S5

IC50 and IC80 values (µg/ml) for pooled sera from C57BL/6 mice injected with Combimab1 either at one site or at three different sites.

Viral strains	IC50			IC80	
	Serum from mice			Serum from mice	
	Injected with Combimab1 at one site	Injected with Combimab1 at three different sites		Injected with Combimab1 at one site	Injected with Combimab1 at three different sites
CE1176	0,017	0,005		0,143	0,110
CH119	0,020	0,023		0,162	0,212
398F1	0,030	0,026		0,127	0,109
TRO11	0,031	0,027		0,207	0,131
BJOX2000	0,245	0,202		1,274	1,252
CE0217	0,051	0,020		0,171	0,161
X2278	0,032	0,067		0,162	0,320
CNE8	0,136	0,191		0,947	1,271
246F3	0,098	0,076		0,459	0,309
X1632	0,020	0,008		0,077	0,045
25710	0,054	0,020		0,411	0,115
